# The Use of Mobile Health to Deliver Self-Management Support to Young People With Type 1 Diabetes: A Cross-Sectional Survey

**DOI:** 10.2196/diabetes.7221

**Published:** 2017-02-15

**Authors:** Rosie Dobson, Robyn Whittaker, Rinki Murphy, Manish Khanolkar, Steven Miller, Joanna Naylor, Ralph Maddison

**Affiliations:** 1 National Institute for Health Innovation School of Population Health University of Auckland Auckland New Zealand; 2 Waitemata District Health Board Auckland New Zealand; 3 Auckland District Health Board Auckland New Zealand; 4 Department of Medicine Faculty of Medical and Health Sciences University of Auckland Auckland New Zealand; 5 Institute for Physical Activity and Nutrition School of Exercise and Nutrition Sciences Deakin University Melbourne Australia

**Keywords:** mHealth, diabetes mellitus, mobile phone, mobile applications, text messages

## Abstract

**Background:**

Young people living with type 1 diabetes face not only the challenges typical of adolescence, but also the challenges of daily management of their health and evolving understanding of the impact of their diagnosis on their future. Adolescence is a critical time for diabetes self-management, with a typical decline in glycemic control increasing risk for microvascular diabetes complications. To improve glycemic control, there is a need for evidence-based self-management support interventions that address the issues pertinent to this population, utilizing platforms that engage them. Increasingly, mobile health (mHealth) interventions are being developed and evaluated for this purpose with some evidence supporting improved glycemic control. A necessary step to enhance effectiveness of such approaches is to understand young people’s preferences for this mode of delivery.

**Objective:**

A cross-sectional survey was conducted to investigate the current and perceived roles of mHealth in supporting young people to manage their diabetes.

**Methods:**

Young adults (16-24 years) with type 1 diabetes in Auckland, New Zealand, were invited to take part in a survey via letter from their diabetes specialist.

**Results:**

A total of 115 young adults completed the survey (mean age 19.5 years; male 52/115, 45%; European 89/115, 77%), with all reporting they owned a mobile phone and 96% (110/115) of those were smartphones. However, smartphone apps for diabetes management had been used by only 33% (38/115) of respondents. The most commonly reported reason for not using apps was a lack of awareness that they existed. Although the majority felt they managed their diabetes well, 63% (72/115) reported wanting to learn more about diabetes and how to manage it. A total of 64% (74/115) respondents reported that they would be interested in receiving diabetes self-management support via text message (short message service, SMS).

**Conclusions:**

Current engagement with mHealth in this population appears low, although the findings from this study provide support for the use of mHealth in this group because of the ubiquity and convenience of mobile devices. mHealth has potential to provide information and support to this population, utilizing mediums commonplace for this group and with greater reach than traditional methods.

## Introduction

Rates of type 1 diabetes are increasing both in New Zealand and internationally [1-4]. Good diabetes control at an early age is integral in delaying the onset and slowing the progression of long-term complications of the disease such as renal failure, blindness, and lower limb microvascular disease [5-8]. Type 1 diabetes is one of the most demanding chronic conditions, both psychologically and behaviorally, requiring considerable daily management throughout the individual’s life for optimal outcome. Successful diabetes management involves managing medical responsibilities (including blood glucose monitoring and insulin administration) alongside behaviors around diet and physical activity [9]. There is evidence of a positive relationship between diabetes self-management behaviors and metabolic control, particularly in adolescence [10-15]. Poor engagement with diabetes self-management in adolescence is likely to continue to poor self-management behaviors in adulthood [16], making this a vital period for the establishment of good self-management and prevention of debilitating long-term complications.

There is a wide range of interventions designed to support young people to self-manage their diabetes that tend to involve providing encouragement, information, and motivation to obtain greater control of their condition. This may be done by increasing their understanding of diabetes, encouraging them to be active participants in decision making around their condition, and motivating them to engage in healthy behaviors [17]. The importance of a combination of approaches for self-management support in individuals with type 1 diabetes is clear, including psychosocial support, education, diabetes monitoring and insulin-specific information and support, and multidisciplinary clinician support [18]. In particular, there is a need for interventions to actively demonstrate the link between diabetes management behaviors and the young people’s daily life [19] and interventions that utilize platforms that engage young people.

Mobile health (mHealth) is the use of mobile devices, such as mobile phones, to deliver health services and information [20]. There is increasing evidence for the effectiveness of mHealth in health behavior change and disease management, including diabetes [21,22]. Mobile phones offer potential to reach all populations and provide access to an individual at opportune times regardless of location [23]. They also provide a tool for support outside of the hospital or clinic and in turn support increased independence. They provide a nonconfrontational method for support around sensitive issues such as contraception, alcohol and drugs, and sexual health, which can have considerable impact on a young person’s diabetes control [24]. In addition, mHealth interventions capitalize on existing communication behavior in young people, who are more likely to bring their mobile phones than their glucose meters to clinic appointments [25].

There is growing evidence that mHealth interventions can successfully engage young people with diabetes, which has traditionally been difficult to do [25-38]. Research indicates strong patient interest in mHealth tools to support diabetes management [39] and a preference for smartphone apps in the young adult population [39]. However, with increasing availability of diabetes-related apps there is concern regarding the accuracy and evidence base of many of these [40]. It is essential to ensure that patients have access to tools that are safe, evidence-based, and known to be effective. In addition, these tools need to attend to the issues relevant to the young person rather than purely focusing on those considered relevant by the clinician [27]. To accommodate the preferences and priorities of the population, and thereby enhance the chances of success, it is crucial to engage the target population in the design and development of the intervention through obtaining feedback during intervention design [41,42] and linking of diabetes management with individual goals and priorities [19,43].

This study aimed to investigate the current and perceived roles of mHealth in supporting young people to manage their diabetes. This will inform the development of a self-management support intervention for this population.

## Methods

### Study Design

A descriptive cross-sectional survey was conducted with young adults with type 1 diabetes from March to September 2014. The survey incorporated both closed and open-ended questions to gain more in-depth information and to allow participants to elaborate further. The survey was designed in paper format and then uploaded into an electronic format using LimeSurvey (LimeSurvey Project), an Open Source survey tool. The survey was then pretested by researchers, members of the study advisory group, and young people. The survey is described according to the CHERRIES (Checklist for Reporting Results of Internet E-Surveys) checklist [44].

### Ethics Approval

Ethical approval for this trial was obtained from the New Zealand Health and Disability Ethics Committee (14/CEN/24). Research approval from each district health board was also obtained.

### Inclusion Criteria

Inclusion criteria were young adults aged 16-24 years (inclusive), diagnosis of type 1 diabetes, registered as a patient under one of the 3 Auckland regional diabetes services, able to read and understand English, and able to provide informed consent.

### Procedures

All patients who met the inclusion criteria were sent a letter from their clinician inviting them to take part in the closed survey. Those wishing to participate could complete the survey via 1 of 3 methods: (1) by going directly to the study website and completing it online, (2) over the phone, or (3) by completing a paper copy of the survey. Participants had 3 months from the letter date to complete the survey.

Before commencing the survey, participants provided informed consent (electronic consent if completing the survey online, verbal consent if completing over the phone, or written consent if completing on paper) to participate. Participants had to enter a unique code from their letter to ensure only those eligible completed the survey. Participants only had access to the survey once their unique code was entered and verified. Upon completion of the survey, participants had the option to enter their contact details to receive a NZ $20 voucher reimbursing them for their time. This personal information was stored separately to the main data file and password protected.

The survey was identical for all participants (no randomized items), and participants were able to go back and change their responses before submission. Adaptive questioning was used to minimize response burden and reduce complexity of questions.

### Survey Design

The survey consisted of 3 parts, with each part of the survey presented on a separate page:

1. Demographic information and technology access: including age, sex, ethnicity, occupation, age at diabetes diagnosis, and mobile phone ownership and use.
2. Using technology to manage diabetes and health: use and perceived usefulness of currently available apps, interest in text message (short message service, SMS)–based diabetes support, and preferences for mHealth content.
3. Your diabetes and how you manage it: perceptions of own diabetes management, confidence in diabetes management, diabetes self-management tasks they find most difficult, and whether they would like to learn more about diabetes and its management.

### Statistical Analysis

Survey data were analyzed and summarized using descriptive quantitative analyses including means, standard deviation, and proportions. Qualitative comments were analyzed using a simple, general inductive thematic approach to identify common themes and meanings from the data. Only completed surveys, with correct unique codes, were included in the analysis and no time limit was imposed. Prioritized ethnicity was used as recommended by the New Zealand Ministry of Health for the reporting of ethnicity data; only one of the ethnic categories nominated by the participant was used according to a predetermined hierarchy (Māori, Pacific Islander, Asian, European, and other ethnic groups, in order of prioritization) [45].

## Results

### Survey Response

There were 141 entries to the survey website; of these, 115 completed the survey, giving a completion rate of 82%. All participants chose to complete the survey online.

### Part 1: Demographic Information and Technology Access

A total of 115 young adults completed the survey, giving a response rate of 29% (see Table 1 for a breakdown of respondents) of the invited population (N=402). There was no significant difference between those invited to complete the survey and those who responded in terms of ethnicity or age. There was a significant difference (*P*=.02) for sex, with the participant sample having a higher proportion of females. The mean age of diagnosis as reported by participants was 11.12 years (SD 5.15, range 1-23).

**Table 1:** Demographics of invited population and survey respondents.

Characteristic		Total eligible (n=402), n (%)		Respondents (n=115), n (%)	
Sex: male		229 (56.9)		52 (45.2)	
**Ethnicity**					
	European	301 (74.9)		89 (77.4)	
	Māori	27 (6.7)		10 (8.7)	
	Pacific Islander	41 (10.2)		7 (6.1)	
	Asian	22 (5.5)		6 (5.2)	
	Other	9 (2.2)		3 (2.6)	
	Not stated	2 (0.5)		0 (0.0)	
Age in years, mean (SD)		19.94 (2.47)		19.51 (2.53)	

All of those who completed the survey reported they owned a mobile phone, with 110/115 (96%) reporting they owned a smartphone (a mobile phone with the addition of a computer operating system). Of those who owned a smartphone, only 71/110 (65%) reported having access to data (Internet) on their phone all the time, 36/110 (33%) sometimes, and 3/110 (3%) reported never accessing the Internet on their phone. The majority of respondents reported that their mobile phone was “pay-as-you-go” (84/115, 73%) as opposed to 30/115 (26%) who were on a monthly contract, and 1 respondent did not know. Most participants (76/115, 66%) reported that they never turn their phone off.

### Part 2: Using Technology to Manage Diabetes and Health

#### Apps for Diabetes Management

A total of 38 (33%) of the 115 respondents reported they use or have used apps to help them manage their diabetes. Rates of app usage differed by ethnicity, with Māori and Pacific Islander respondents having lower rates of app use (10% and 0%, respectively) compared with Europeans (38%), Asians (33%), or the other ethnicities (33%). A total of 43% of females who completed the survey reported they use or have used apps to help them manage their diabetes compared with 21% of males.

Of those who reported having used apps (n=38), they reported having used a mean of 1.87 different apps (SD 1.33, range 1-7). The most commonly reported apps were Carbs & Cals (n=10), Glucose Buddy (n=9), MyFitnessPal (n=6), and DAFNE Online (n=5). Of the 38 respondents who had used apps, 10 (26%) reported finding them “extremely useful” in helping them manage their diabetes, 20 (53%) reported finding them “a little useful,” and 8 (21%) “not very useful.” Common reasons included that they were useful for tracking diabetes data, for carbohydrate counting and insulin calculations, and that they were accessible and convenient for managing diabetes on the go. Participants reported that a key benefit of the apps was that they provided an easy and convenient way to track their diabetes and store data. They also reported that they provided an easy way to see patterns through graphs of their capillary glucose data.

Helps me keep a record of what I'm eating my levels and my insulin intake along with the other medications I'm on and shows a pattern of when I go lower or higher

They graph patterns of blood sugar levels, and are a great place to store data related to your diabetes

They reported that, because their phones are always with them, they provided a great way to manage their diabetes on the go. They mentioned having access to information when out as being a benefit and providing a replacement for pen and paper methods of keeping track of their diabetes.

Because I always have my phone and it makes it easier to log in my blood sugars on something that I always have access too

I find them useful because I'm always on the go and I always have my smartphone on me so if I ever need to check the carb content of something I can do so immediately

A number of participants also reported that apps are useful for carbohydrate counting, particularly for less common foods or meals, and for calculating insulin dosage:

It [the app] gives me an idea of how many carbs are in foods I don't normally consume on a day to day basis. Takeaway foods in particular are harder to count carbs in.

It makes the maths easier- calculating how much insulin I need when taking into account how many carbs I'm eating and what my current blood glucose is.

The most common reason for why they were not useful was the data entry being tedious and therefore not being bothered to use the app.

Too fiddly to mess around with and enter all the data. As well as when I test my blood, I just want to eat not mess around with my phone.

Just another thing to remember to do so I can’t be bothered sometimes.

Technical issues were also identified as a reason for apps not being perceived as useful, and the need for Wi-Fi or data connection meant they could not always use them.

Sometimes I can find out how many carbs my food I'm eating has but I have trouble using it sometimes because it doesn't load and it requires internet access and if I haven't got good reception I can't see the portion sizes

Others reported that because they forget to use the app or do not enter their data regularly the app was not useful.

I fail to enter in my readings from my testing machine. So therefore I have no data in the app at all.

Only 1 participant identified not being able to trust the app and another identified cost as a barrier.

I didn't really trust them, I was too scared to use them in case they were wrong

The majority (33/38, 87%) of the participants who had used apps reported that they would recommend diabetes-related apps to other young people with diabetes. Those who had never used apps to manage their diabetes were asked to provide the reasons. The most common reasons for not having used apps to help manage their diabetes included that they did not know of any apps or that they existed at all, that they did not feel they would be of any help or use to them, or that they did not feel that they needed them.

#### Text Messages for Diabetes Management

A total of 74 (64%) of the 115 respondents reported that they would like to receive SMS text messages designed to support them to manage their diabetes. Those who were interested were asked how often they would want to receive messages, with 21% of the 74 wanting messages more than once per day, 17% once per day, 15% only once every few days, and 12% once a week or less. They were also asked to identify topics they would want the SMS text messages to be about (see Table 2), with tips on how to manage diabetes being the most preferred topic.

**Table 2:** Topics for text messages (n=74).

Topics	Count^a^	Percentage of sample, %
Tips on how to manage my diabetes	57	77
Motivational messages	50	68
Reminders to test my blood glucose	45	61
Information about diabetes	38	51
Other^b^	7	10

^a^Participants could identify multiple topics.

^b^Other topics identified by participants included recreational drugs, interesting diabetes facts, and research updates.

The 41 participants who reported that they would not be interested in receiving SMS text messages to support them were asked to provide their reasons. Key responses included they did not think that they needed them and concern that they would be annoying. There were 19 participants who identified that they did not feel they needed SMS text message–based diabetes support as they felt they were already managing their diabetes well.

Because I feel I'm perfectly capable of managing my diabetes myself, I know what i have to do and know what I face if i am neglectful. Someone texting me with things I already know is unnecessary.

I think it's unnecessary and have done fine on my own for the most part.

In addition, 19 of the 41 participants reported they would not sign up as they felt that it would be annoying and would just remind them that they had diabetes when they did not want to think about it.

I think I manage my diabetes quite well, I don't really need to read motivational messages and I check my blood glucose regularly on my own. I personally can find texts like that can become annoying. I may consider using the option only if there was an unsubscribe option available if I didn't want to receive them anymore.

Will become irritating. Sometimes it is nice to forget, [believe] that you aren’t diabetic - having constant reminders will continuously remind you that you are different from everyone else

There were 2 participants who felt that this type of support would not be personal enough and 2 participants who reported they would prefer apps to SMS text messages.

### Part 3: Diabetes and How It Is Managed

A majority (66/115, 57%) of the respondents reported that they felt they managed their diabetes “extremely” or “very” well. There were 44/115 (38%) who reported that they managed their diabetes “not so well” and only 5/115 (4%) “not well at all.” On a scale from 0 “Not at all confident that I can manage my diabetes” to 10 “Completely sure I can manage my diabetes,” participants reported a mean rating of 7.23 (SD 2.091, range 1-10). Significantly lower ratings of confidence were seen in those who reported interest in receiving SMS text messages designed to support them to manage their diabetes (mean 6.89, SD 2.10) than those who did not (mean 7.85, SD 1.94; *P*=.02). Although more than half the participants reported that they felt that they managed their diabetes well, 72/115 (63%) reported that they would like to learn more about diabetes and how to manage it. Participants were also asked about the 3 specific areas of diabetes management that they find most difficult; results are presented in Table 3. The most common areas identified were “Remembering to check my blood glucose” (55/115, 48%) and “Eating well” (40/115, 35%).

**Table 3:** Diabetes self-management tasks participants find most difficult (n=115).

Diabetes self-management tasks	Count	Percentage of sample, %
Checking my blood glucose	35	30.4
Remembering to check my blood glucose	55	47.9
Eating well	40	34.8
Managing insulin	27	23.5
Problem solving (especially around blood glucose, highs and lows, sick days)	29	25.2
Being psychically active	31	27.0
Attending my medical appointments	21	18.3
Other^a^	10	8.7

^a^Other responses included remembering to administer insulin, needles, carbohydrate counting, being in unexpected situations unprepared, correcting highs, managing alcohol, recording blood glucose levels, and the social effects.

## Discussion

### Principal Findings

This study aimed to investigate the perceived role mHealth can play in supporting young people to manage their diabetes and to inform the development of a self-management support intervention for this population. Overall, results indicated interest in mHealth for supporting diabetes self-management and provides further support for mobile phones to deliver self-management support in this population group because of high access. In addition, the survey highlighted that although young adults were confident in their ability to manage their diabetes, there was strong interest in learning more about aspects of diabetes management.

As expected, mobile phone, and in particular smartphone, ownership in this population was high, although more than one-third of the respondents did not have consistent access to Internet or data on their device. Because of the demanding and continuous nature of diabetes self-management, tools to support this group need to take data access into consideration. A downside of many of the currently available apps is the need for the user to have ongoing Internet or data to access many of the apps’ functionalities. Apps designed to be used offline, therefore avoiding the need for ongoing data or Internet access, can require greater storage capacity on the phone to download the app and this can be a barrier for those with lower-level devices that typically have smaller storage capacity.

Contrary to expectations, use of currently available apps was low in this group, particularly in Māori and Pacific Islander respondents. The lack of awareness of available apps as well as a perception that these would not be of use contribute to the low utilization of the most accessible mHealth tools for this population. The use of apps by participants for insulin calculations is of concern in light of research showing that most insulin calculation apps could be putting patients at risk of harm by providing no protection for incorrect insulin dosage recommendations [40]. A common use of apps in this study was for the collection and tracking of data, which, owing to the members of this population commonly having their phone with them and turned on at all times, is ideal. The privacy of this information was highlighted in a recent letter in the *Journal of the American Medical Association* on the lack of privacy policies in diabetes apps [46]. They reported that 81% of the diabetes apps investigated did not have privacy policies and, of those that did, many of the provisions did not actually protect the user’s privacy. Health care professionals have the potential to play a key role in increasing the awareness of apps and recommending that patients choose guideline-based and secure apps to increase safety.

A strong interest in the use of SMS text messaging for diabetes self-management support was observed, particularly among those with lower confidence to manage their condition. Previous research has shown that as beliefs in the ability to maintain a healthy lifestyle increased, the need for support through SMS text messaging decreased [47]. Therefore, designing SMS text messaging interventions for those with lower confidence in their ability to manage their condition may be of greater pertinence and more positively received than for those already confident in their ability to manage their diabetes.

Of particular interest to participants was the use of SMS text messaging for providing motivation, reminders, and diabetes self-management tips. Nearly half the respondents reported that remembering to test their blood glucose was the part of their diabetes management that they found most difficult. Although the use of continuous glucose sensors, which have the option of setting high and low alerts, provides a solution, cost is currently a major barrier to widespread use of this technology [48]. Therefore, SMS text messaging is an attractive option to provide testing reminders because of the instant delivery as well as the low patient cost and high accessibility and reach of this type of mHealth tool [49].

This study highlights key factors that need to be considered when designing SMS text messaging–based diabetes self-management support, including the potential for messages to be annoying, to be unnecessary, or to not be personal enough. Therefore, future development of tools for this group needs to be tailored and personalized, rather than a “one size fits all” approach. Although it has been previously reported that young adults with diabetes have differing priorities from their health care team, our results indicate that nearly two-thirds were interested in learning more about diabetes. mHealth could provide the ideal medium for supporting learning as it can be personalized, nonconfrontational, and delivered at the time and place that it is needed and wanted.

Several limitations of the study should be noted. The response rate, although largely reflective of the wider population, was low, limiting the generalizability of the results. It is likely that those who did respond may have more interest in mHealth and therefore actual engagement with this type of tool may be even lower in the wider population. All respondents to the survey completed it online with none requesting to complete it by paper or phone. Although the alternatives were offered, it may be that those without Internet access were less likely to take part, biasing the sample to a more technological group. In addition, the self-report and cross-sectional design of the study are key limitations, as is the sex distribution of the sample differing from the target population. Although this study investigated the use of apps in this population, it did not assess how the population was using them and for how long. It is important that research into mHealth tools for this population assesses the degree of engagement, including intensity and duration, to ensure tools are designed to meet their needs and improve outcomes.

### Conclusions

This study provides valuable insight into the engagement of young adults with type 1 diabetes with currently available mHealth tools, as well as providing insight into how future mHealth interventions can be designed to meet their need. The input of the end users regarding their use and preferences for mHealth tools provided by this survey will allow for the development of a more relevant and a potentially more efficacious intervention.

## Abbreviations

CHERRIES: Checklist for Reporting Results of Internet E-Surveys
mHealth: mobile health
SMS: short message service
